# Legal and scientific deficiencies of drug advertisements on German television

**DOI:** 10.1007/s00210-024-03604-8

**Published:** 2024-11-15

**Authors:** Malin Philipp, Roland Seifert

**Affiliations:** Institute of Pharmacology, Hannover Medical School, Carl-Neuberg-Str. 1, Hanover, D-30625 Germany

**Keywords:** Drug advertising, Therapeutic Products Advertising Act (Medicines Advertising Law), Medicinal Products Act, Television advertising

## Abstract

Drug advertising primarily serves to publicize the product and increase sales. The legal requirements in the Therapeutic Products Advertising Act (Heilmittelwerbegesetz, HWG) in Germany are intended to protect consumers. However, a lack of information about the potential negative aspects of drugs represents a risk for viewers. In this study, we analyzed a total of 52 advertising clips for drugs, food supplements, and medical products on public and private German television. These short films in the commercial breaks between the TV programs serve to publicize and advertise products. Furthermore, the corresponding package inserts for the drugs and the content and structure of the advertising clips were analyzed. The clips were compared with each other as well as about the mandatory information according to §4 of the HWG and §11 of the Medicinal Products Act (Arzneimittelgesetz, AMG). The study revealed that a large proportion of the mandatory information was not included in the advertising clips analyzed. Not all legal requirements were met in the package inserts either. Alarmingly, the advertising clips consistently contained only one of the mandatory information pieces, namely, the name of the product. Adverse effects were not mentioned in any advertising clip, and the effects of drugs were only poorly explained, if at all. Overall, scientific content of the advertisements was minimal if at all present. Instead, the airtime was dominated by emotional and attention-grabbing content in the form of a frame story, whereas the potential to educate people about medicines and raise public awareness remains mostly unused. Thus, drug advertisements on German television exhibit substantial scientific and legal deficiencies. In addition, we noted several legal gray zones in advertisements. The deficiencies in drug advertisement quality on television are, unfortunately, in line with quality deficiencies on drug advertisements in various other platforms (see., e.g., https://pubmed.ncbi.nlm.nih.gov/37792047/; https://pubmed.ncbi.nlm.nih.gov/38430230/). Thus, the lack of rigorous implementation of the HWG is a general problem in Germany. We make specific suggestions on what actions should be taken to improve the implementation of the HWG in television advertisements to improve drug safety and consumer safety.

**Supplementary Information:**

The online version contains supplementary material available at 10.1007/s00210-024-03604-8.

## Introduction

In pharmaceutical advertising, a distinction is made between specialist advertising and audience advertising. Audience advertising means advertising to the general public. In contrast to this, specialist advertising refers only to specialized personnel, which includes doctors and pharmacists who are allowed to prescribe these medicines themselves or trade in them legally (Vögtli 2021; Ewert 2020). One form of audience advertising is television advertising. In German television, private television channels are distinguished from public television channels. Public channels include ARD and ZDF, while examples of private channels include RTL, SAT1, and PRO7. While advertising on public channels is limited to 20 min per weekday, advertising on private channels may not exceed 20% of daily airtime (Mandelmond, 2023; Solmecke 2015).

Advertising on public television channels may only be broadcast until 8 pm. After that, official prime time begins in Germany, which ends at 11 pm. However, the period from 5 pm to 8 pm is also very important. Many Germans use television after work to wind down at the end of the working day, so there is already talk of a new primetime (Gans 2024).

According to the Bundeszentrale für politische Bildung (Federal Agency for Civic Education) (2016), advertising primarily serves to raise awareness of products, ideas, or services by increasing sales. An emotional component is often used to influence the audience’s purchasing behavior and motivate them to buy the advertised products and services. Advertising is also intended to enhance the image of the advertising product by highlighting the positive aspects compared to competing products. By repeating the advertising message several times, there is an effect on memory and learning processes. In addition to all these functions, advertising should also serve as a source of information by highlighting product features, qualities, uses, and prices.

Requirements for drug advertising on television are set out in the HWG (Bundesministerium der Justiz 2023). Alongside the Law against unfair competition (Gesetz gegen den unlauteren Wettbewerb, UWG) and the professional regulations, it forms the legal framework for advertising in the German healthcare sector and comprises a total of 18 paragraphs. The original version dates back to 11.07.1965, and the last amendment was made on 19.07.2023. It contains various prohibitions and bans. For example, misleading advertising is prohibited. This includes claiming therapeutic efficacy that does not exist as well as creating the impression that success can be expected with certainty or that no harmful effects can occur. In addition, advertising may only be organized for competitive purposes. Advertising for unapproved drugs is prohibited, as is the advertising of prescription drugs outside specialist circles, including for example doctors and pharmacists. The package insert represents the basis for the information in television advertising for medicinal products. The mandatory information for the package insert is also laid down in §11 of the AMG (Bundesministerium der Justiz 2022).

To the best of our knowledge, drug advertisements on German television have never been analyzed scientifically. The current study was intended to show how extensively the legal requirements for drug advertising and package inserts are complied with or disregarded to develop concepts on how education for drugs on television could be improved. The following questions were elaborated:

1. To what extent are the legal requirements for drug advertising on television complied with?
2. To what extent are the legal requirements for drugs complied with in the corresponding package inserts?

This also gave rise to the following questions:

1. What is the structure of drug advertising clips?
2. To what extent is attention paid to patient education in the form of explaining the drug’s effects through drug advertising?
3. Which stylistic devices are used to make advertising more attractive to viewers or potential customers (for instance, visual metaphors, symbolism, dialog, and narrative structures)?

## Material and methods

The analysis includes a total of 52 advertising clips that were broadcast on German television in the period from 09.03.2022 to 16.08.2022. Private (RTL, SAT1, PRO7, VOX, RTLII, and sixx) and public television channels (ARD and ZDF) were involved. The advertising clips were collected at random with the aim of collecting an equal number of advertising clips on private and public television channels. The focus was on collecting a large number of different advertising clips. The airtime of the advertising clips was not taken into account for the analysis. In addition to 34 advertising clips for drugs, 12 advertising clips for food supplements and 6 for medical products were analyzed as well (Figure S1).

At the beginning of the recording process, the commercial breaks on television were filmed by smartphones. Afterward, the advertising clips were then cut out of the recordings. After a short time, we found that the majority of the advertising clips could be found online on YouTube, so these were used as basis for the analysis with a clear quality advantage. Marginal differences between the advertising clips broadcast on television and the advertising clips uploaded to YouTube were classified as irrelevant. Austrian and Swiss advertising clips were used as well if a German version was not available online. These did not differ in content but only in the display of the warning “In case of risks and adverse effects, read the package insert and ask your doctor or pharmacist.” While German advertising is required by law to display this sentence separately from the advertising content in white lettering on a gray background, the postscript was sometimes integrated directly into the advertising in foreign advertisements. Furthermore, the postscript was not included in all advertising clips available online. However, it was not possible to check retrospectively whether it was already missing during the broadcast on television or was merely removed when the advertising clip was uploaded to YouTube. Due to this discrepancy, the postscript was excluded from the analysis. A timeline was created for each advertising clip at the beginning of the analysis, in which the content of the advertising clip was displayed over time.

In addition to the advertising clips, the accompanying package inserts for the advertised drugs were analyzed, as basis for the information that should be included in the advertising clips. The following mandatory information was analyzed in the package inserts: pharmaceutical company, manufacturer, indication, active ingredients, dosage, effect, adverse effects, interactions, preparation form, and instructions for taking and use (Table S1). The following mandatory information was examined in the advertising clips: pharmaceutical company, name of the medicinal product, active ingredients, indication, adverse effects, and labeling if a preparation is available only on prescription (Table S2).

In addition to the mandatory information, the duration of the advertising clips and the duration of the frame story were also examined. As frame story, the content without pharmacological significance whose removal from the advertisement would not result in a loss of information was summarized. This included, for example, dialogs between the protagonists, shots of nature, or creative introductions for the advertising clip. The duration was documented in seconds and was set in relation to the total length of the respective advertising clip. It was examined whether reference was made to the effect of the drug in the clip. For this purpose, it was analyzed whether the explanation of the effect was auditory or visual or whether it was displayed in small print. In addition, the duration and quality of the explanation of the effect were examined. The quality was classified into five categories (--, -, 0, +, or ++). Furthermore, the following contents were examined: Interactions, preparation form, instructions for taking and use, protagonists’ own experiences, recommendations, and prominent actors (Table S3).

**Fig. 1:**
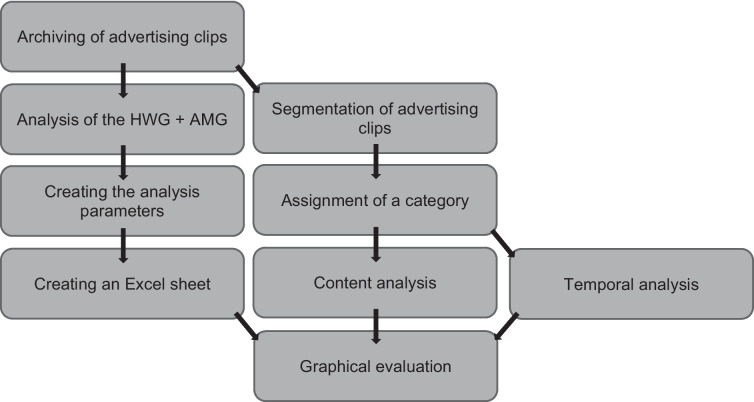
Graphical representation of the methodological approach

To analyze not only the content of the advertising clips but also their structure, all 52 clips were broken down into ten segments based on their duration (10%- increments). Each segment was assigned a category. The nine categories that emerged from the analysis are the following: frame story, product introduction, indication, slogan, promise of effectiveness, packaging, effect, other product, and recommendation (Table S4). The categories were analyzed in the advertising clips in terms of both content and timing. With the content analysis, we examined how the individual segments are composed and which categories are generally found in the segments (Figure S2-S11). In the temporal analysis, we looked at the occurrence of the categories developing over the course of the ten segments and in which segment the categories were primarily represented (Figures S12-S20). Figure 1 shows a schematic representation of the methodological approach.

## Results

### Distribution of advertising clips on the examined television channels

**Fig. 2:**
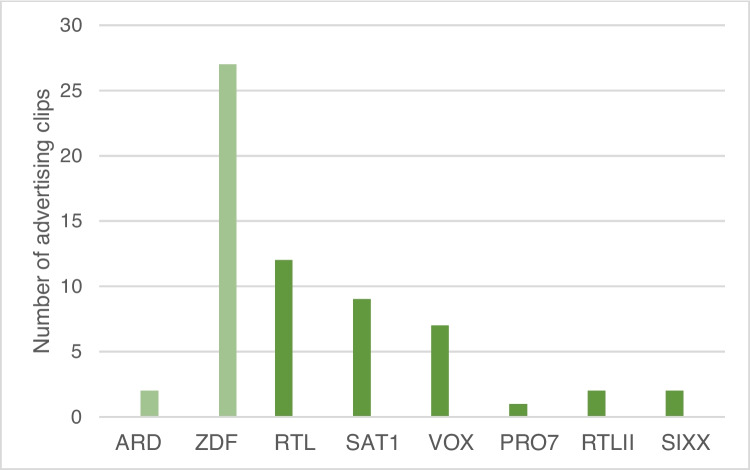
Distribution of advertising clips on the examined television channels. In light green, public television channels. In dark green, private television channels

A total of 52 advertising clips were analyzed. The advertising clips were taken from public and private television channels in roughly equal proportions; some of the advertising clips were found in both channel categories (Fig. 2). The assignment of an advertising clip to a television station means that the advertising clip was first discovered on this channel. It does not mean that the advertising clip can only be found on this television channel.

### Indications for use of the investigated drugs

**Fig. 3:**
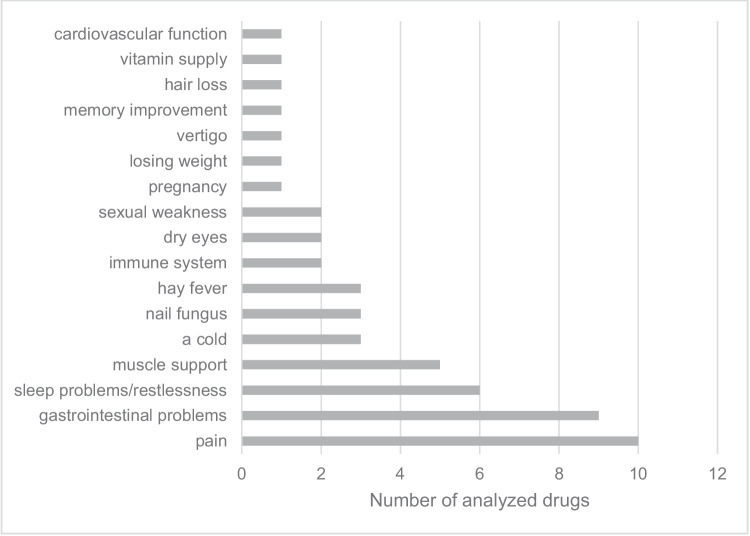
Grouped indication range according to the package insert

The four most frequently mentioned indications in the package insert were pain, gastrointestinal problems, sleep problems or restlessness, and muscle support (Fig. 3). These strongly dominating complaints are typically found in elderly persons. The advertised painkillers mainly include non-steroidal anti-inflammatory drugs (NSAIDs; cyclooxygenase inhibitors) such as ibuprofen, acetaminophen (paracetamol), and combination preparations.

### Compliance with the mandatory information in the package insert according to §11 of the AMG

Table 1 shows an overview of compliance with the examined mandatory information in the package inserts. In 98% of the package inserts, the pharmaceutical company was stated. The manufacturer’s details were complete in 79%. An indication was given in 92% of the package inserts. The active ingredients, dosage, and preparation form were stated in 100% of the package inserts. The effect was explained in 44% of the package inserts. The adverse effects were stated in 62% of the package inserts, and in 13%, the information was unclear. The interactions were mentioned in 33% of the package inserts, in a further 33%, the information was unclear. Directions for taking and use were included in all package inserts.

**Table 1:**
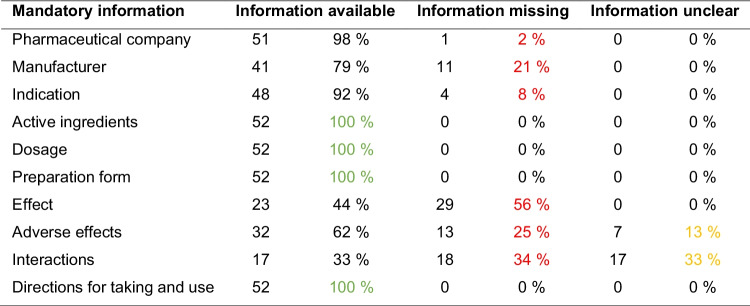
Compliance with the mandatory information in the package insert according to §11 of the AMG. In red, cases with missing mandatory information. In yellow, cases with unclear information. In green, mandatory information included in every package insert

### Compliance with the mandatory information in the advertising clip in accordance with §4 of the HWG

Table 2 shows an overview of compliance with the mandatory information examined in the advertising clips. The information for the pharmaceutical company was complete in one advertising clip. In 75%, it was missing, and in 23%, the information was unclear. The name of the medicinal product was present in all advertising clips. The indication was stated in 58%, and in 21%, the information was unclear. The contained active ingredients were named in 46%; in one case, the information was unclear. Adverse effects were not mentioned in any advertising clip. Similarly, none of the drugs were declared as prescription-only, which should be seen as positive in this case, as the advertising of prescription-only drugs outside specialist circles is prohibited in Germany.

**Table 2:**
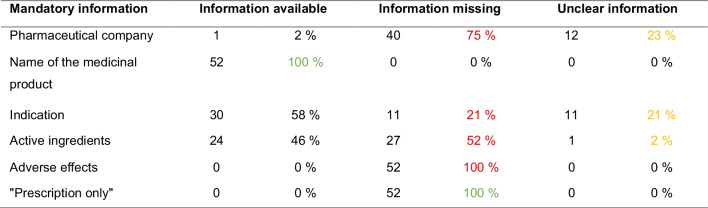
Compliance with the mandatory information in the advertising clip according to §4 of the Therapeutic Products Advertising Act. In red, cases with missing mandatory information. In yellow, cases with unclear information. In green, mandatory information included in every advertising clip

### Investigation of information that were examined in the advertising clip but are only mandatory in the package insert

In addition to the mandatory information for advertising clips, the information or recognizability of the preparation form as well as the effect, interactions, and instructions for taking or use were examined based on the mandatory information in the package insert (Table 3). Although the information is not mandatory for advertising clips, the preparation form and effect were stated in about half of the advertising clips. On the other hand, interactions and instructions for taking and use were only included in a few cases.

**Table 3:** Information that was examined in the advertising clip but is only mandatory in the package insert

Information	Information available		Information missing		Unclear information	
Preparation form	26	50%	7	13%	19	37%
Effect	21	40%	31	60%	0	0%
Interactions	1	2%	50	96%	1	2%
Instructions for taking and use	7	13%	45	87%	0	0%

### Examination of the duration of the advertising clips

**Fig. 4:**
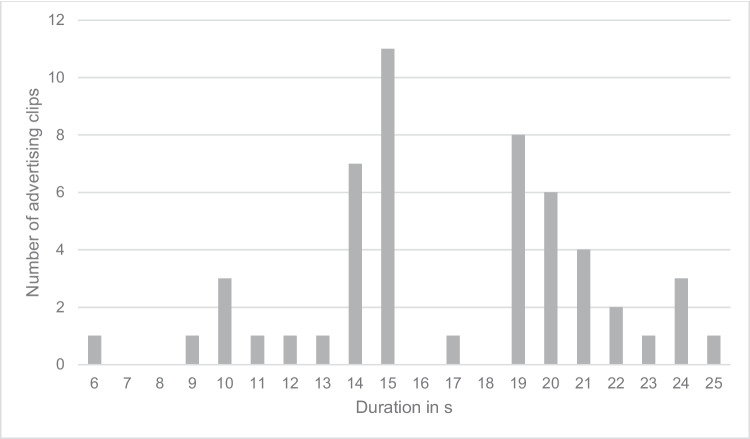
Duration of the advertising clips in seconds

The shortest advertising clip analyzed lasted 6 s, and the longest 25 s (Fig. 4). The average length was 17 s, and the median was 15.5 s. We did not observe a normal distribution of the clips. Rather, we identified a population of shorter clips (up to 15 s) and a population of longer clips (up to 25 s).

### Investigations into the explanation of the effect of the drug

**Fig. 5:**
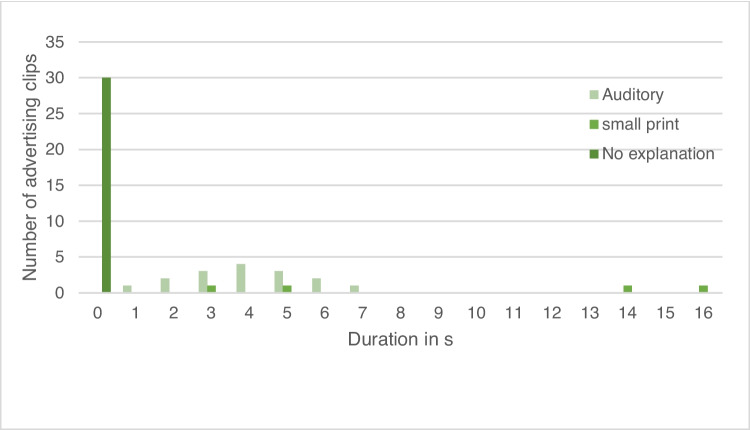
Duration of the explanation of the effect of the drug in the advertising clips

In 30 of the 52 advertising clips analyzed, the duration of the explanation of the effect of the drug was 0 s (Fig. 5). In 16 advertising clips, the effect of the drug was referred to auditorily, and the average duration of the explanation of the effect of the drug was four seconds. In four other advertising clips, the explanation of the effect of the drug was displayed in small print. In two of the advertising clips, the explanation of the effect of the drug was displayed for an average of 4 s, and in the other two for an average of 15 s.

**Fig. 6:**
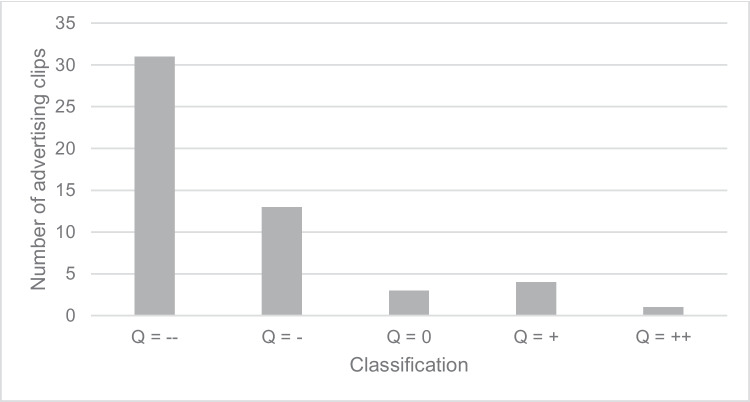
Classification of the quality of the explanation of the effect of the drug into five categories

Figure 6 shows the classification of the quality of explanation of the effect of the drug in the advertising clips in five categories. No reference was made to the effect in category “--.” If an impact target of the drug was named, category “-” was assigned. In category “0,” the effect of the drug was explained superficially. If the effect of the drug was explained in detail, category “+” was assigned. In the “++” category, the effect of the drug was both explained in detail and presented visually. Of 52 advertising clips analyzed, 31 fell into the “--” category, as no reference was made to the effect. The best category “++” could only be awarded in one case. Four advertising clips were awarded the “+” category and three advertising clips the “0” category. The remaining 13 advertising clips were classified in the “-” category.

### Examination of the frame story

**Fig. 7:**
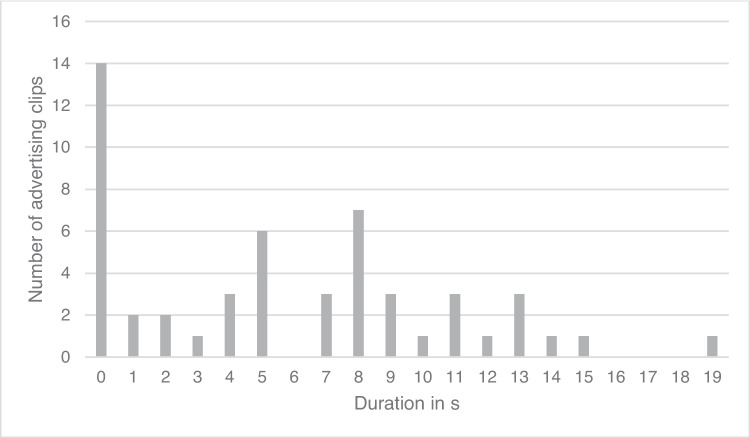
Duration of the frame story in seconds

**Fig. 8:**
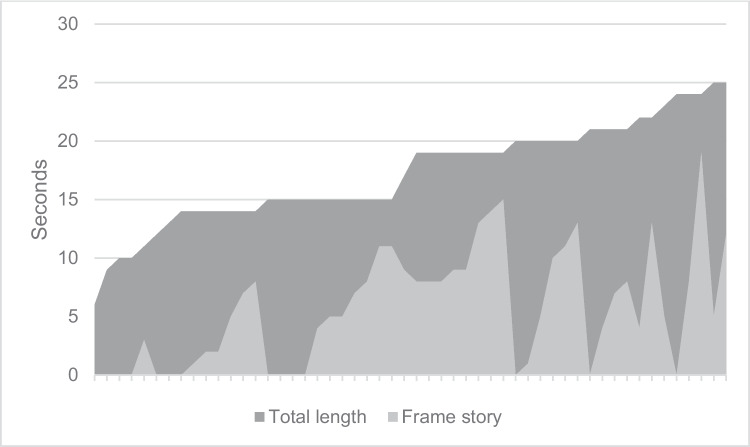
Frame story in relation to total length

In 14 of the 52 advertising clips analyzed, there was no frame story (Fig. 7). In the remaining advertising clips, the duration of the frame story lasted between 0 and 19 s. The average duration was 7.8 s, and the median 10 s. Figure 8 shows the temporal relation between the total length and duration of the frame story.

### Segmentation of advertising clips

Figure 8 shows an overview of the structure of the advertising clips examined. It shows the ten segments into which each advertising clip was broken down and the categories from which each segment is made up. The first segment consists of the frame story in 71% of the advertising clips. Other advertising clips begin primarily with the naming of the indication or the product presentation. Up to the fifth segment, these remain the three main categories. The occurrence of the frame story decreases steadily, while the product presentation appears more frequently. The proportion of explanations of the effect increases up to the sixth segment and then decreases again. The second half of the advertising clips is primarily made up of the categories promise of effectiveness, packaging, slogan, and recommendation.

## Discussion

### Poor compliance with the legally mandatory information in the package insert and advertising clip

There are ten mandatory disclosures for the package insert. The analysis showed that only four of these ten mandatory details were provided in full in each of the package inserts examined (Table 1). These included the name of the active ingredients, the dosage, the preparation form, and the directions for take and use. The information of the pharmaceutical company (one exception) and the indication (four exceptions) were almost complete. In contrast, there were large gaps in the information on the manufacturer, the effect, the adverse effects, and the interactions. It is noticeable that the latter is primarily information that could pose major risks to consumers if missing. The statement in some package inserts that no interactions were known should also be viewed critically, as this did not mean that these had been investigated.

Six mandatory disclosures for the advertising clips were examined as part of the study (Table 2). The information also includes a reference if a medicine is subject to prescription. However, prescription-only medicines may only be advertised to doctors, dentists, veterinarians, pharmacists, and persons who are authorized to trade in these medicines (HWG, §4). This therefore excludes advertising to the general public on television and also explains that there were no prescription-only medicines among the medicines examined. Consequently, this information was omitted from the advertising clips and the ban was complied with. Of the remaining five mandatory statements analyzed, only one, namely, the name of the drug, was fulfilled in every advertising clip. With regard to the pharmaceutical company, the indication, the active ingredients, and the adverse effects, most of the information was missing, incomplete, or too specific.

Keuper and Seifert (2023) investigated compliance with the legal requirements for pharmaceutical advertising in the German magazine Apotheken Umschau. Advertisements for 123 preparations were analyzed. The results of this study are comparable to the results for television advertising (Table 4) with regard to the mandatory information that applies to both print and television advertising. The mandatory information is also not fully complied with. Only the name of the medicinal product was stated in every advertisement in both Apotheken Umschau and television advertising. The indication is almost completely stated in the Apotheken Umschau. The pharmaceutical company and the composition were stated more frequently than on television, although adverse effects were only mentioned in one case in the Apotheken Umschau.

**Table 4:**
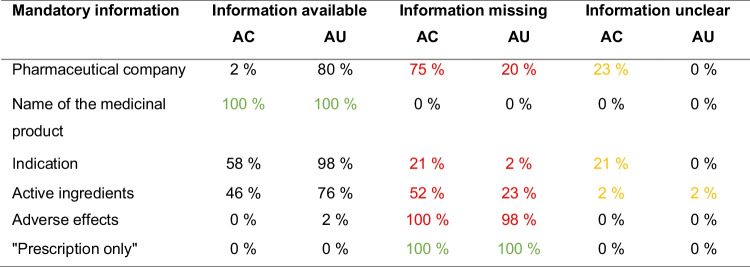
Direct comparison between compliance with the mandatory information in the advertising clips (AC) and in the Apotheken Umschau (AU) (according to Keuper and Seifert, 2023). In red, cases with missing mandatory information. In yellow, cases with unclear information. In green, mandatory information included in every advertising

Two other studies also came to similar conclusions when examining compliance with the HWG. Kuschel and Seifert (2024) examined drug advertising in the Deutsche Apotheker Zeitung (German Pharmacy Journal), an independent journal addressed primarily to pharmacists. A total of 167 advertisements for non-prescription medicines were analyzed. Similarly, there were major information gaps, such as a lack of references to adverse effects. The study by Barlage and Seifert (2024), which was focused on compliance with legal requirements in the product catalogs of two different online pharmacies, showed the analogous results. In conclusion, the inadequate compliance with the law is not only a problem in television advertising, but in print advertising for the general public, advertising in specialist circles, and online pharmacies as well.

In view of the fact that all of the drugs examined are not prescription-only and therefore can be used without any control by a doctor, it is particularly clear from the predominant lack of information on interactions and instructions for taking and use that patient education and objective information is not the focus of drug advertising. This is also emphasized by the fact that the font size of the information displayed is often minimized to the point of illegibility or information such as the form of the preparation is neither mentioned nor displayed, but can at best be read from the packaging shown. Although every drug is accompanied by a package insert as a legally required information carrier, which is intended to serve as a generally understandable summary of the most important information for the consumer (Blasius 2014), a study by Bruchmann and Nägelein (2018) showed that the package insert is rarely consulted in reality. The study was conducted by Promio.net on behalf of Nebenwirkungen.de and investigated whether and how Germans deal with package inserts and the adverse effects of medicines. A total of 1535 men and women aged between 25 and 60 took part in the study across Germany. The study showed that only a quarter of respondents always read the package insert for prescribed medicines, and only one in five for over-the-counter medicines. Almost one in ten stated that they never read the package insert for over-the-counter medicines. The categories “adverse effects,” “dosage,” “instructions for use,” and “indications” were mentioned most frequently when the study participants were asked what they would read in the package insert for prescription medicines. Nevertheless, around half of all respondents stated that they did not feel sufficiently informed about adverse effects. The study suggested that many patients felt that the instructions for use were too long and too complicated. This becomes a cause for concern when the adverse effects of non-prescription drugs are underestimated.

The lack of external information is becoming a problem, especially in light of the fact that more and more Germans are purchasing their medication from online pharmacies. According to a study conducted by the market research institute Appinio in November 2020 (Appinio 2020), in which a total of 1000 people from Germany aged between 16 and 65 were surveyed, 69% of respondents stated that they at least occasionally order over-the-counter medicines online. Twenty-nine percent of respondents even use online pharmacies for over-the-counter medicines more frequently than local pharmacies, and 7% stated that they would only use online pharmacies. According to the study, 17% of online pharmacy users did so for the first time during the coronavirus pandemic, primarily among 18- to 34-year-olds. The main advantages of online pharmacies are their convenience but also the fact that the product range can be viewed very easily and prices can be compared. The disadvantages, on the other hand, are the lack of advice and external control over whether customers are aware of adverse effects and interactions.

A 2014 report by the Regensburger Verbund für Werbeforschung (Regensburg Association for Advertising Research) (Mitteilungen des Regensburger Verbunds für Werbeforschung 2014), based on empirical studies on the advertising of prescription drugs, described how advertising can lead to increased health worries among potential consumers and ultimately to an increase in the unnecessary use of medication. In addition to the direct functions of informing and motivating, advertising also fulfills the function of socialization. For example, the use of certain products should be made “socially acceptable” and implemented as normal behavior. In this way, a lack of information about the adverse effects of medication also harbors the risk of trivialization. Without information, advertising also encourages self-treatment through the listing of symptoms and the presentation of the drug as a solution to these complaints in the course of self-diagnosis. This involves the risks of overmedication and overdosing. Furthermore, adverse effects and interactions can easily be underestimated by people without the appropriate specialist knowledge. One of the package inserts examined included the information that the medication should only be used under medical supervision. In view of the fact that the drugs analyzed are only over-the-counter medicines, it would certainly make sense for the consumer to receive this information prior to purchase, whether from a pharmacist or a warning displayed in the advertising clip. It can be concluded that only a small proportion of the mandatory information in the advertising clips examined is complied with and that the information in the accompanying package inserts is also incomplete in some cases, which poses a risk to the consumer in several respects.

### Structural design of the advertising clips

The examination of the structural composition of the advertising clips made clear that the frame story plays a dominant role in pharmaceutical advertising (Fig. 7). The frame story has no pharmacological-medical added value in terms of information, so the relations between this content and the overall length of the advertising clips were analyzed. Not every advertising clip contained an explicit frame story, but there were also many advertising clips in which the frame story took more than half or even three-quarters of the advertising time (Fig. 8). It is noticeable, however, that both the shorter and the longer advertising clips tend to have less frame story. The largest proportion of frame story is found in the commercials with a medium duration.

Against the background of the average duration of an advertising clip of 17 s, the average duration of the frame story of 6 s seems quite long. If the advertising clips without a frame story are excluded from the analysis, the average duration of the frame story increases to 8 s, which corresponds to almost 50% of the advertising time. This result reinforces the assumption that the function of advertising as a source of information tends to be neglected. This can also be seen in the analysis of the explanation of the effect in the advertising clips (Figs. 5 and 6). The average duration of the explanation of the effect is only 1.2 s. The analysis of the quality of the existing explanation of the effect also makes clear that the focus is not on informing viewers and potential consumers, even though this is not a mandatory requirement for the advertising clips but only for the package inserts. Where an explanation was included, in most cases, only the impact target was mentioned, but no further reference was made to the operating principle (Figure S20). Of 52 advertising clips, the effect of the advertised medication was only explained in detail and comprehensibly in five cases. Of these, only one advertising clip stood out both in terms of its auditory explanation of the effect and its visual presentation.

In an issue of Media Perspektiven (4/2004), the ARD Research Service presented a number of studies on the subject of advertising design and its effect on radio and TV commercials. In one of these studies, which dealt with attention-grabbing design elements in TV commercials, Doormann (2003) summarizes that an advertising clip must contain three different factors to generate attention. These included activation, involvement, and likeability. Viewers need to be psychologically activated (for example, through intense auditory and visual stimuli or emotional stimuli that act as “eye-catchers”) and involved internally so that the advertising clip is ultimately viewed with favor. A comprehensible plot, suspense, aesthetics, emotionality, and entertainment value are important for this. Another empirical study (Trommsdorff and Becker 2003) examined the significance of creativity for the effectiveness of advertising clips. The more creativity criteria (for example, originality, clarity, and inventiveness) were fulfilled in the advertising clip, the higher its effectiveness, particularly with regard to achieving psychological goals (for example, product awareness, product image, and awareness).

**Fig. 9:**
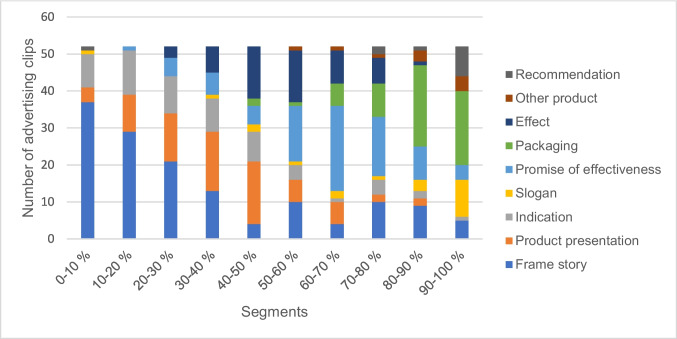
Composition of the individual segments at a glance

The results of these two studies therefore underline the importance of the dominance of the frame story in the structure of the advertising clips. Furthermore, it is clear that various design elements are used to make advertising appear authentic and enable personal identification (Fig. 9). This was evident, for example, in the frequency of personal experiences (Figure S21) shared by the protagonists of the advertising clips. This design element was used in more than a quarter of all advertising clips. In addition, a quarter of all commercials also included a personal recommendation from satisfied users or emphasized the market leadership of the drug in German pharmacies (Figure S22).

It also became apparent that drug advertising is designed with a focus on appealing to the viewer. The frame story, product presentation, and promise of effectiveness stand in the foreground; the function of informing and educating is rather neglected. This also becomes clear once again in Table 5. The most important key features with regard to pharmacological content are summarized here. There may also be a correlation with the average age of viewers. A study published by the Statista Research Department (2011) showed that the average age of viewers on ZDF and ARD was 60 in 2011. By contrast, the average age of the private television channels surveyed in 2011 was between 35 and 51. A study conducted by AGF Videoforschung in collaboration with GfK showed that the average age in 2022 was already 64 for ARD and 65 for ZDF (Mantel 2022). The average age of viewers of private television channels has also risen to 44 to 54 years, but there is still a clear age difference between private and public television channels. The selection of medicines advertised, which mainly refer to pain, gastrointestinal complaints, and sleep problems and therefore primarily correspond to the health restrictions of the older generation, also speaks for the interpretation of drug advertising for an older audience.

**Table 5:** Overview of the key features of the advertising clips

Number	Indication	TV channel	Duration in s	Frame story in %	Mandatory information complied with (max. 6)
1	Hair loss	SAT1	19	68	3
2	Gastrointestinal complaints	VOX	19	47	2
3	Immune system	RTL + ZDF	22	59	3
4	Sleep problems	RTL	19	42	3
5	Immune system	VOX	25	48	2
6	Pain	RTLII	15	73	2
7	Cold	VOX	15	47	3
8	Gastrointestinal complaints	VOX	14	57	3
9	Restlessness/sleep problems	SAT1 + ZDF	21	38	3
10	Dry eyes	VOX	12	0	3
11	Gastrointestinal complaints	SAT1	15	0	3
12	Cold	VOX	20	55	4
13	Cold	RTL	15	27	3
14	Muscle support	SAT1 + ZDF	15	33	3
15	Muscle support	ZDF	15	33	3
16	Nail fungus	RTL	24	33	3
17	Hay fever	SAT1	11	27	3
18	Pregnancy	RTLII	19	42	4
19	Weight loss	ZDF	25	20	3
20	Headache	RTL + ZDF	22	18	3
21	Hay fever	RTL	14	7	3
22	Pain	RTL + ZDF	23	22	4
23	Sleep problems	VOX + ZDF	20	5	4
24	Hay fever	PRO7	9	0	2
25	Support muscles, bones, blood clotting	ZDF	20	50	4
26	Nail fungus	RTL + ZDF	14	36	3
27	Sexual weakness	ZDF	14	0	3
28	Muscle support	SAT1	19	74	4
29	Gastrointestinal complaints	SAT1	21	19	4
30	Gastrointestinal complaints	SAT1	19	47	3
31	Nerve pain	ZDF	10	0	3
32	dizziness	ZDF	14	14	3
33	Memory improvement	RTL + ZDF	21	33	3
34	Restlessness/sleep problems	ZDF	13	0	2
35	Intestinal complaints	ARD	20	0	3
36	Intestinal complaints	ZDF	15	0	3
37	Pain	ZDF	19	42	2
38	Restlessness	SIXX	14	14	4
39	Muscle/nerve support	SAT1	24	0	3
40	Pain	SIXX	15	0	4
41	Sexual weakness	ARD	19	79	2
42	Pain	ZDF	15	73	3
43	Nail fungus	ZDF	10	0	3
44	Pain	ZDF	15	53	3
45	Pain	ZDF	14	50	3
46	Sleep problems	ZDF	21	0	4
47	Dry eyes	RTL	20	25	4
48	Vitamin supply	ZDF	17	53	3
49	Intestinal complaints	ZDF	15	0	3
50	Pain	ZDF	24	79	2
51	Cardiovascular function	ZDF	20	65	3
52	Intestinal complaints	ZDF	6	0	3

### Limitations of the study

The analysis was limited to 52 current advertising clips, and we could not go back to history since previous clips were not accessible to us. A high-quality version of advertising clips on YouTube was not available for all items analyzed. It could not be guaranteed that the broadcast version on television and the version analyzed were identical. Due to the use of Austrian and Swiss advertising clips and correspondingly different legal requirements, the mandatory warning in Germany “In case of risks and adverse effects, read the package insert and ask your doctor or pharmacist.” could not be included in the analysis. In individual cases, there were incorrect information in the package insert or discrepancies between the package insert and the advertising clip. When asked, not all pharmaceutical companies or manufacturers responded, meaning that these discrepancies could not be corrected for the analysis. However, as the analysis performed here only examined whether a statement was made and not which one, this has no effect on the results. The package inserts used as the basis for the analysis were found online. In some cases, no online versions were available but were sent by the pharmaceutical company or manufacturer on request. For those package inserts that were available online, it was not possible to check whether they were the most up-to-date version. Some of the rating scales developed in this study are not completely objective but have a subjective character.

Lastly, the authors have a medical and pharmacological background and no formal legal education but did their best to assign the advertisement content to proper legal categories. An argument for the validity of this paper comes from the fact that so far five independent primary investigators (and accordingly, first authors; including this study) analyzed various aspects of drug advertisements and came to similar conclusions (Keuper and Seifert 2023; Leemhuis and Seifert 2024; Barlage and Seifert 2024; Kuschel and Seifert 2024).

## Conclusions

Despite legal requirements, the mandatory information for drug advertising in television was largely disregarded. The naming of negative properties of the advertised drugs was avoided. Instead of professional information, the focus laid on emotional and attention-grabbing content in the form of a frame story, which dominated the main part of the advertising clips. As television advertising reaches a very broad audience, drug advertising could be used to raise awareness and educate the public, which would involve more than just the reference “For risks and adverse effects, read the package leaflet und ask your doctor or pharmacist.”

However, this potential has so far remained largely untapped. The purpose of the HWG is to protect consumers, which means that the enforcement of the law should also be monitored more closely and more strictly. Urgent action is needed to improve the quality of drug advertisements on television and to rigorously enforce the HWG. In line with our study, a recent article in the daily newspaper Süddeutsche Zeitung complained that viewing drug advertisements on German public television is “painful” (https://www.sueddeutsche.de/medien/werbung-gesundheit-medikamente-1.6332186?reduced=true). From a scientific perspective, unfortunately, we must support the journalistic conclusions.

Along the same line, it will be most informative to study drug advertisement on television in other countries with different legal frameworks.

### How to improve drug advertisements on television: drug companies, television channels, and BfArM all must act

Given the fact that television reaches and influences millions of people daily and that many drug advertisements are broadcasted at “prime time,” e.g., in the early evening before the evening program starts, urgent action by all stakeholders regarding drug advertisements in television must act. Below are some suggestions that can be easily implemented with *bona fide* effort:

- Even though an emotional component is important to attract the viewer’s attention, the study showed that, as a result, the scientific information content suffers in most cases. But very few advertisements delivered scientific content. Thus, science can be transported even in a television commercial. The prototypes, although very rare, are available.
- For many mandatory information, it would not be a challenge to integrate them into the advertising clip. As has already been shown in some commercials, for example, the name of the pharmaceutical company can be displayed with the help of a logo without losing airtime. The name of the active ingredient or the dosage can be displayed, and the risk of interactions can be briefly mentioned.
- The aim of the advertisement should not only be to market the product in a positive way but also to inform viewers about the product, including the risks. Broadcast time, which is largely used for the frame story, could be filled with information about the indication, effect, and ingredients, as demonstrated in some commendable advertising clips. An advertising clip does not necessarily need a compelling frame story, but consumers do need someone to confront them with the dangerous aspects of a drug. In the long run, this will increase trust in the drug and drug company.
- Television channels have responsibility as well. Particularly, the large public television channels have sufficient staff with a medical and legal background who could easily check the quality of the submitted advertisements based on the criteria laid out in the HWG and applied in this paper and other studies from our group (Keuper and Seifert 2023; Barlage and Seifert 2024; Kuschel and Seifert 2024; Leemhuis and Seifert 2024). Television channels must not shy away from rejecting low-quality advertisements simply because of being afraid to lose drug companies as customers. Trust of the viewers in the validity and integrity of broadcast content is the most valuable (social) capital that television channels have. Poor quality content leads to a loss of this capital.
- Lastly, the Bundesinstitut für Arzneimittel and Medizinprodukte (BfArM, The Federal Institute for Drugs and Medical Devices) is responsible for supervising the proper implementation of the Heilmittelwerbegesetz (HWG, Therapeutic Products Advertising Act; Medicines Advertising Law). BfArM must start issuing hefty penalties for violations of the law. As has been shown in this study and related studies (Keuper and Seifert 2023; Barlage and Seifert 2024; Kuschel and Seifert 2024; Leemhuis and Seifert 2024), violations of the HWG are very abundant and encompass various types of drugs. The violations include, among others, phytomedicines and homeopathic medicines, and concern heterogenous advertisement platforms, ranging from traditional journals for consumers and professional journals to online pharmacy platforms and public and private television. In many cases, including those presented here, drug companies exploit gray zones (labeled in yellow color in Tables 1, 2, and 4) to present their products in a legally ambiguous manner. Thus, the lack of proper implementation of the HWG in Germany is a general problem that has not been solved for decades (Keuper and Seifert 2023; Barlage and Seifert 2024; Kuschel and Seifert 2024; Leemhuis and Seifert 2024). Strict implementation of the law will increase drug safety and consumer safety and will benefit society in its entity.
- In view of the fact that many gray areas of the HWG are being exploited, a revision of the law would also be conceivable. For example, the font size in which information and risks are displayed should be increased to ensure legibility or a minimum duration for the display of the active ingredient could be specified. Generally applicable warnings for the broadcasting of pharmaceutical advertising could also be included in the legal requirements.

## Supplementary Information

Below is the link to the electronic supplementary material.

Supplementary file1 (DOCX 331 KB)

## Data Availability

All source data for this study are available upon reasonable request.

## Abbreviations

HWG: Heilmittelwerbegesetz (Therapeutic Products Advertising Act; Medicines Advertising Law)
UWG: Gesetz gegen den unlauteren Wettbewerb (Law against unfair competition)
AMG: Arzneimittelgesetz (Medicinal Products Act)
