# Supplementary material for: Legal and scientific deficiencies of drug advertisements on German television
Source: Naunyn Schmiedebergs Arch Pharmacol. 2024 Nov 15;398(5):5287–301. doi: 10.1007/s00210-024-03604-8 (PMC11985658; doi:10.1007/s00210-024-03604-8)
Supplement: Supplementary file 1 — Supplementary file1 (DOCX 331 KB) [file 210_2024_3604_MOESM1_ESM.docx]

**Supplemental tables S1-S4 and figures S1-S22**

**Malin Philipp and Roland Seifert**

**Legal and scientific deficiencies of drug advertisements in German Television**

**Table S1:** Mandatory information examined in the package inserts

| Mandatory information | Explanation |
| --- | --- |
| Pharmaceutical company  Manufacturer  Indication  Active ingredients  Dosage  Effect  Adverse effects  Interactions  Preparation form  Instructions for taking an and use | Who is named as the pharmaceutical company?  What is the name of the manufacturer?  Which disease pattern is to be alleviated by the medication?  Which active ingredients are included?  How much active ingredient is contained per unit (e.g. tablet)?  How does the medicine work? Which substance or indication group is named?  What adverse effects can occur? How often do they occur?  What interactions are described?  Which preparation form is mentioned?  What information can be found on how to take or use the medicine? How long should the medicine be taken for? How should the medicine be dosed? |

**Table S2:** Mandatory information examined in the advertising clips

| Mandatory information | Explanation |
| --- | --- |
| Pharmaceutical company | Is the name/company name and registered office of the pharmaceutical company stated? Is a logo displayed? |
| Name of the medicinal product  Active ingredients  Indication  Adverse effects  Subject to prescription | Is the name of the medication mentioned or displayed?  Is it stated which active substances are contained? If yes, is the information complete?  Which disease pattern is to be alleviated by the medication? Was the indication sufficiently stated or was the information incomplete or too specific?  Are adverse effects mentioned?  Was the note "prescription only" displayed? |

**Table S3:** Information in the package inserts examined in addition to the mandatory information according to § 4 HWG

| Category | Explanation |
| --- | --- |
| Duration | How many seconds does the advertising clip last (minus the postscript "For risks and adverse effects, read the package insert and ask your doctor or pharmacist")? |
| Effect | Is the effect of the drug described? |
| Interactions | Are interactions described? |
| Preparation form | Is it recognizable which preparation form is involved? If not, is it legible on the packaging shown? |
| Instructions for taking and use | Is information provided on how to take or use the product? |
| The protagonists' own experiences | Do protagonists promote drugs by talking about their own experiences and recommendations? |
| Recommendations | Is it stated what percentage of users are satisfied or is the "No. 1" award mentioned? |
| Prominent actors | Do prominent actors appear in the advertising clip? |

**Table S4:** Analyzed categories in the segmentation of the advertising clips

| Category | Explanation |
| --- | --- |
| Frame story | Introductory words, dialog, introduction of the protagonists, nature shots, etc. |
| Product presentation | First presentation of the medicinal product (examples: Handing over a tube of ointment, presentation of the medicinal product as a solution for the disease pattern) |
| Indication | Naming the required symptoms/the disease pattern |
| Slogan | catchy phrase that summarizes the advertising message |
| Promise of effectiveness | Specification of the desired effect |
| Packaging | Presentation of the drug together with the packaging |
| Effect | Explanation of the effect |
| Other product | Advertising another medicinal product of the same brand |
| Recommendation | E.g. "89 % of users are satisfied" or "No. 1 in German pharmacies" |

**Figure S1:** Classification into medicinal products, food supplements and medical devices

**Figure S2:** Percentage distribution of categories in the first segment

**Figure S3:** Percentage distribution of categories in the second segment

**Figure S4:** Percentage distribution of categories in the third segment

**Figure S5:** Percentage distribution of categories in the fourth segment

**Figure S6:** Percentage distribution of categories in the fifth segment

**Figure S7:** Percentage distribution of categories in the sixth segment

**Figure S8:** Percentage distribution of categories in the seventh segment

**Figure S9:** Percentage distribution of categories in the eighth segment

**Figure S10:** Percentage distribution of categories in the ninth segment

**Figure S11:** Percentage distribution of categories in the tenth segment

**Figure S12:** Distribution of the category frame story across all segments

**Figure S13:** Distribution of the category product presentation across all segments

**Figure S14:** Distribution of the category indication across all segments

**Figure S15:** Distribution of the category slogan across all segments

**Figure S16:** Distribution of the category promise of effectiveness across all segments

**Figure S17:** Distribution of the category packaging across all segments

**Figure S18:** Distribution of the category effect across all segments

**Figure S19:** Distribution of the category other product across all segments

**Figure S20:** Distribution of the category recommendation across all segments

**Figure S21:** Advertising with personal experiences of the protagonists in the advertising clips

**Figure S22:** Mention of a recommendation or "No. 1"
